# Development of a fully human anti‐GITR antibody with potent antitumor activity using H2L2 mice

**DOI:** 10.1002/2211-5463.13451

**Published:** 2022-06-21

**Authors:** Qiuli Tong, Hu Liu, Qianqian Qi, Chaohui Dai, Teddy Yang, Feng Qian

**Affiliations:** ^1^ Shanghai Public Health Clinical Center, Human Phenome Institute and School of Life Sciences Fudan University Shanghai China; ^2^ Shanghai Chempartner Co., Ltd China; ^3^ Shanghai PharmaExplorer Co., Ltd. China

**Keywords:** engineering modification, fully human antibody, GITR, H2L2 mice, immunization strategies, T cell

## Abstract

Glucocorticoid‐induced TNF receptor‐related (GITR) can act as a co‐stimulatory receptor, representing a potential target for safely enhancing immunotherapy efficacy. GITR is triggered by a GITR ligand or an agonist antibody and activates CD8^+^ and CD4^+^ effector T cells, reducing tumor‐infiltrating Treg numbers and resulting in activation of immune responses and tumor cell destruction by effector T cells. GITR is an attractive target for immunotherapy, especially in combination therapy with immune checkpoint inhibitors, as is being explored in clinical trials. Using H2L2 transgenic mice encoding the human immunoglobulin variable region and hybridoma technology, we generated a panel of fully human antibodies that showed excellent specific affinity and strong activation of human T cells. After conversion to fully human antibodies and engineering modification, we obtained an anti‐GITR antibody hab019e2 with enhanced antitumor activity in a B‐hGITR MC38 mouse model compared to Tab9H6V3, an anti‐GITR antibody that activates T cells and inhibits Treg suppression from XenoMouse. As a fully human antibody with its posttranslational modification hot spot removed, the hab019e2 antibody exerted more potent therapeutic effects, and may have potential as a novel and developable antibody targeting GITR for follow‐up drug studies.

## Graphical abstract

As a costimulatory molecule, GITR is a potential target for safely enhancing immunotherapy efficacy. GITR triggered by agonist antibodies may provide the desired biological outcome of inhibiting Treg while activating T effector cells. A novel fully human anti‐GITR antibody hab019e2 from H2L2 mice shows good agonistic activity *in vitro* (A) and potent therapeutic effects *in vivo* (B).

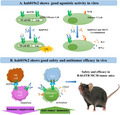

**Abbreviations**
CynoGITR: cynomolgus GITR
FACS: fluorescence‐activated cell sorting
GITR: glucocorticoid‐induced TNF receptor‐related
GITRL: GITR ligand
hGITR: human GITR
IFN‐γ: interferon‐gamma
MFI: mean fluorescence intensity
NF‐ĸB: nuclear factor (NF)‐kappa B
NK: natural killer
PBMCs: peripheral blood mononuclear cells
TNFRSF: tumor necrosis factor receptor superfamily
Treg: regulatory T cell
VH: variable region of heavy chain
VL: variable region of light chain

As a tumor necrosis factor receptor superfamily (TNFRSF) member, glucocorticoid‐induced TNF receptor‐related (GITR) shares significant homology in its intracellular domain structure with a subgroup of TNFRSF members that include CD27, OX40, and 4‐1BB and has similar and to some extent different biological functions [1, 2, 3]. GITR is constitutively expressed on CD4^+^CD25^+^ regulatory T cells, and its expression has also been confirmed to be upregulated by specific activated T cells [4, 5, 6]. Moreover, it is involved in controlling T‐cell‐mediated responses, including organ‐specific autoimmunity chronic infection and antitumor immunity [7, 8]. GITR ligand (GITRL) is expressed at low levels in B cells, macrophages, and dendritic cells (DCs) [9]. The GITR/GITRL pathway has been confirmed to upregulate DCs function and promote T‐cell‐mediated immunity [10].

Ligation of GITR on immune cells by GITRL/agonist antibodies had profound effects on kinase phosphorylation, surface receptor expression, suppressive activity, and cytokine production [11]. Stimulation of GITR on effector CD8^+^ T cells results in high‐avidity T‐cell responses to tumor‐specific antigens, thereby inducing potent antitumor immunity in the absence of autoimmunity [8]. GITR signaling, mediated by agonist antibody, can limit effector T‐cell sensitivity to Treg suppression and increase its antitumor response *in vitro* and *in vivo* [12, 13].

Based on the potent preclinical antitumor activity of agonist anti‐GITR antibodies, GITR represents a potential target for enhancing the efficacy of immunotherapies [14, 15, 16]. The combination of a GITR antibody with the anti‐PD‐1 antibody pembrolizumab, especially in patients with melanoma who are insensitive to treatment, has been shown to have good safety and potential activity [17]. In mouse tumor models, the combined application of a GITR antibody and a CTLA‐4 antibody results in 80% tumor inhibition, reduction of intra‐tumoral Treg (via GITR), and stimulation of CD8^+^ T cells (via CTLA‐4) [18, 19]. It has been also reported that GITR antibodies combined with vaccines can achieve synergistic and complementary antitumor effects in cervical cancer and melanoma [20, 21]. Moreover, the addition of chemotherapy or gemcitabine, to this combination of a vaccine and a GITR monoclonal antibody, reduces the tumor‐suppressive environment and induces a persistent memory immune response [22].

To obtain fully human therapeutic antibodies, transgenic animal immunization with antigens producing human antibodies is the most successful method to date. The H2L2 mouse line is a ‘second generation’ transgenic mouse engineered by Harbor to produce antibodies with human V‐region chains and rodent constant regions. Harbor's H2L2 mouse features an immune response comparable to normal mice while offering diverse human V‐gene usage, so they produce antibodies with diversified and mature affinity through endogenous affinity maturation and immune effector function. The H2L2 platform facilitates the rapid generation of antibodies and converts lead candidates into clinical development without undergoing time‐consuming steps such as humanization or affinity maturation. Combining Harbor's H2L2 mouse with hybridoma technology provides a superior platform for fully human GITR therapeutic antibody discovery [23].

Presently, there are no fully human GITR antibodies on the market, and only a few antibodies are in clinical trials [24]. Here, we used an H2L2 transgenic mouse line which represents an alternative source of human antibodies *in vivo* and hybridoma technology, we obtained four candidates. After posttranslational modification hot spot removal, hab019e2 and hab070e1 were assessed for potential *in vivo* efficacy in the B‐hGITR MC38 mice, and hab019e2 demonstrated good therapeutic value and safety.

## Materials and methods

### Cells and recombinant proteins

The human GITR gene, encoding its ECD (extracellular domain; amino acids Gln26‐Glu161, Thr45Ala, UniProt#Q9Y5U5.1), or cynoGITR gene encoding ECD (Gln20‐Glu155, NCBI #XP_005545180.1), were cloned into the pCPC vector containing the human Fc gene, followed by transient transfection into FreeStyle™ 293F cells (Invitrogen, Carlsbad, CA, USA; #R79007) for recombinant protein production. The GITR antibody Tab9H6v3 (9H6v3; AMGEN INC., Thousand Oaks, CA, USA; US 2015/0064204 Al) and Tab6C8 (6C8; GITR, Inc., Cambridge, MA, USA; US 8388,967 B2) were generated internally based on sequences in patents as reference antibodies. GITR protein and antibodies purified by protein A were fully characterized by affinity and activity.

The nucleotide sequences that encode the full‐length amino acid sequence of human GITR (NCBI #NP_004186.1) or cynoGITR (NCBI #XP_005545180.1) were cloned into the pLVX‐IRES vector (Clontech, Mountain View, CA, USA), and then packaged into lentiviral particles. GITR overexpression stable cell lines (293F‐hGITR, 293F‐cynoGITR, and Renca‐hGITR) were generated by lentivirus infection and validated with the reference antibodies. Stable high GITR overexpression subcloned cells were used for immunogen expression, screening, and functional identification. A Jurkat‐hGITR‐NF‐ĸB‐Luc cell line was generated by infecting the Jurkat‐NF‐ĸB‐Luc cell line with lentiviral particles, and stable selection for a higher ratio of induction with the reference antibody compared to the isotype control in reporter assays.

### H2L2 mice and immunization

Different immunization strategies were used to immunize 6‐ to 8‐week‐old H2L2 mice housed under pathogen‐free conditions to obtain better specificity and higher titer affinity. For protein immunization, 50 μg of hGITR ECD hFc protein immunogen with complete Freund's adjuvant (Sigma #F5881; St. Louis, MO, USA) was used to immunize each mouse intraperitoneally for initial immunizations, and then 25 μg of protein with incomplete Freund's adjuvant (Sigma #F5506) was used for the first boost 2 weeks after initial immunization, followed by another boost 3 weeks after the previous immunization. For cell immunization, Renca‐hGITR cells treated with 10 μg·mL^−1^ mitomycin for 1 h or 293F‐hGITR cells were used to immunize mice at 5 × 10^6^ cells/500 μL per mouse, and the schedule of immunization was similar to that for protein immunization. For gene gun immunization, H2L2 mice were immunized four times using a Helios Gene Gun (Bio‐Rad, Hercules, CA, USA; #165–2431), and each immunization injection was at four spots, with a total of 4 μg of pCPC plasmid encoding the full‐length human GITR (NCBI #NP_004186.1). A subsequent boost immunization was given at subsequent 2‐week intervals.

All of the blood samples were collected 1 week after immunization, and antibody titers and specificity of serum were determined using FACS.

### Generation of human‐murine chimeric antibody

When sufficient specific antibody titers were reached in the serum, mice with a superior immune response to hGITR were selected for fusion with Sp2/0‐Ag14 mouse myeloma cells (ATCC #CRL‐1581) by a high‐efficiency electrofusion method. Hybridomas were then selected and supernatants from the resulting clones were screened by a FACS binding assay and an NF‐ĸB reporter assay. The desired monoclonal hybridoma clone cells were expanded for production. Human‐murine GITR chimeric antibodies were then purified from hybridoma cell culture supernatant using Protein A chromatographic columns (GE Healthcare, Fairfield, CT, USA; #17‐1279‐03) for characterization. The name of each human‐murine antibody was defined by the item number with the initial characters ‘mAb’.

### Protein‐based binding assay

Protein‐based ELISA binding assays followed an indirect ELISA protocol [25], and antigen protein was used to pre‐coat high binding 96‐well ELISA plates, which were incubated with antibody mixtures, and then assessed using an anti‐ratIgG/hIgG‐peroxidase antibody. In T‐cell activation, the cytokine IFN‐γ secreted by activated T cells in the cultured supernatant was measured using the Human IFN‐γ ELISA Kit (R&D System, Minneapolis, MN, USA; #DY285) following the protocol from the manufacturer.

### Cell‐based binding by FACS

The antibodies in supernatants or from purification were tested for affinity to human/cynoGITR protein on the surface of overexpression cells (293F‐hGITR or 293F‐cynoGITR) by FACS. Cells were incubated with antibodies at 4 °C for 1 h in 96‐well plates, and after washing, further incubated at 4 °C for 1 h with fluorescent dye conjugated anti‐rat/human antibodies. The mean fluorescence intensity (MFI) of anti‐GITR antibodies binding to cells was assessed by flow cytometry. The EC50 of the fitting curve was calculated using graphpad prism (GraphPad software, San Diego, CA, USA) software.

### NF‐κB reporter assays

For determining the capacity of antibodies to induce the NF‐ĸB signaling pathway in Jurkat cells overexpressing NF‐ĸB‐Luc and human GITR (Jurkat‐hGITR‐NF‐ĸB‐Luc cell), the cells were seeded at 5 × 10^4^ cells/well in 50 μL of cell assay medium (RPMI1640 + 5% FBS) in 96‐well assay plates (Corning, Corning, NY, USA; #3903). Plates were then incubated with 50 μL of a dilution series of GITR antibodies in the non‐crosslinking format, or 50 μL of a dilution series of F(ab')2 Fragment anti‐Human IgG, Fcγ fragment specific (Jackson Immunoresearch, West Grove, PA, USA; #109–006‐008) and GITR antibody at a ratio of 4 : 1 in the crosslinking format. After incubation at 37 °C in 5% CO_2_ for 5 h, 100 μL of ONE‐Glo™ Luciferase Assay System Reagent (Promega, Madison, WI, USA; #E6120) was added to each well, and luminescence values were measured using a Varioskan LUX (Thermo, Carlsbad, CA, USA).

### T‐cell activation assays

Total T cells from frozen human peripheral blood mononuclear cells (PBMCs, AllCells, Alameda, CA, USA) were first isolated using the EasySep™ Human T Cell Isolation Kit (Stemcell Technologies, Vancouver, BC, USA; #17951). Next, a gradient dilution of antibody solutions was added to each plate (Corning #3599) pre‐coated with a mixture of 0.3 μg·mL^−1^ anti‐CD3 antibodies and 2.7 μg·mL^−1^ anti‐hFc antibody, and then plates were incubated for 30 min. A total of 3 × 10^5^ T cells/well were placed in each plate and then cultured in a carbon dioxide incubator at 37 °C for 3 days. Culture supernatants were then collected for cytokine IFN‐γ secretion level detection using the Human IFN‐gamma DuoSet ELISA kit (R&D #DY285B).

### VH/VL sequencing and cloning

Based on antigen‐binding and functional activity, 42 clones of desired antibodies were selected for variable region sequencing. mRNA from hybridoma cell pellets was extracted using the RNeasy Plus Mini Kit (Qiagen, Hilden, Germany, #74136), and total cDNA was synthesized with the SMARTer® RACE 5′/3' Kit (Clontech #634858). Next, cDNA containing the whole variable region was cloned into a pMD18‐T plasmid and sequenced. Based on the DNA sequences of these variable regions, the amino acid sequences of the variable regions of the chimeric antibodies were used to generate a phylogenetic tree relative to the antibodies from patents.

### Fully human antibodies and engineering modification of lead candidate antibodies

According to our sequencing results, the VH region or mutant VH sequence of anti‐GITR antibodies was recombined into the pCPC expression vector containing a signal peptide and the constant region of human heavy chain hIgG1. The VL region or mutant VL sequence of the anti‐GITR antibody was recombined into the pCPC vector containing the signal peptide and the constant region of human Kappa light chain antibody. Then, the two expression vectors were transiently co‐transfected into FreeStyle™ 293F cells for the production of fully human antibodies or engineering modification candidates. The purified anti‐GITR antibodies were obtained from culture supernatants, and the name of each human antibody was defined by the corresponding antibody item number with the initial character ‘hab,’ and further characterized for affinity and function as indicated previously, and the *in vivo* efficacy was assessed by follow‐up studies.

### Antibody affinity measurement by Biacore

The binding affinity of candidates to hGITR was assessed using the Biacore™ T200 SPR system (GE Healthcare). The antigen hGITR ECD hFc was immobilized on the chip surface after a Series S CM5 sensor chip was activated. Each antibody was diluted in running buffer to a series of concentrations (0, 3.125, 6.25, 12.5, 25, and 50 nm), loaded onto the biosensor for an association time of 180 s, and then the buffer flow was maintained for dissociation for 600 s. The K_*D*_ value for each antibody was evaluated using biacore t200 evaluation software 3.0.

### GITR engineering modification of lead candidate antibodies for mouse experiments

The *in vivo* binding of the anti‐GITR antibody was evaluated in the humanized B‐hGITR knocked in mice model (B‐hGITR mice, Biocytogen, Beijing, China). Dissolve 15 μg anti‐mCD3 antibody (BD Biosciences, Franklin Lakes, NJ, USA; #553294) in 400 μL PBS, and B‐hGITR mice were injected intraperitoneally with 200 μL anti‐mCD3 antibody solution. Twenty‐four hours later, the mice were euthanized and their spleens were quickly extracted to make single‐cell suspension. The spleen cells with erythrocyte cells lysised were resuspended in 300 μL PBS with 6 μL anti‐Mcd16/32 antibody (Biolegend, San Diego, CA, USA; #101310) to block the FcR, and then incubated with PE‐anti‐mCD19 (Biolegend, #115508), PerCP‐anti‐mTcRβ antibody (Biolegend, #109228), or GITR antibodies conjugated FITC to be stained. Fluorescence intensity was measured by FACS.

The *in vivo* antitumor efficacy of anti‐GITR antibodies was evaluated in B‐hGITR MC38 transplanted mice model. The murine colon carcinoma cell line MC38 (Purchased from Shunran Shanghai Biotechnology Co., Ltd., Shanghai, China) was inoculated into the right flank of 24 B‐hGITR mice at 5 × 10^5^ cells in 0.1 mL of buffer. When each tumor grew to approximately 100 mm^3^, the mice were randomly divided into four groups with six mice in each group, including a Tab9H6v3 group and hIgG1 group as controls, and hab019e2 group and hab070e1 groups as sample groups. All of the groups were given an intraperitoneal injection at a dose of 10 mg·kg^−1^, once every 3 days for consecutive 16 days, and the experiment ended 2 weeks after the last administration. Tumor volume and body weight were measured twice a week, and the data were recorded. At the end of the experiment, the animals were euthanized, the tumors were stripped, weighed, and photographed, and the relative tumor inhibition rate (TGI%) was determined.

All mice in the experiments were conducted in the laboratory animals at ChemPartner in compliance with regulations Animal Welfare Assurance and were approved by Institutional Animal Care and Use Committee of ChemPartner (IACUC protocol no.: B11‐201806200001‐20,210,620).

## Results

### Fully human and engineering modification antibodies against human GITR

In this study, H2L2 mice with sufficient antibody titers after being immunized with different strategies were selected for fusion to produce multifarious human‐murine chimeric antibodies against GITR. The clones with binding affinity for human/cynoGITR and agonist activity in NF‐ĸB assays were identified, and 40 of them were selected for sequencing.

**Fig. 1:**
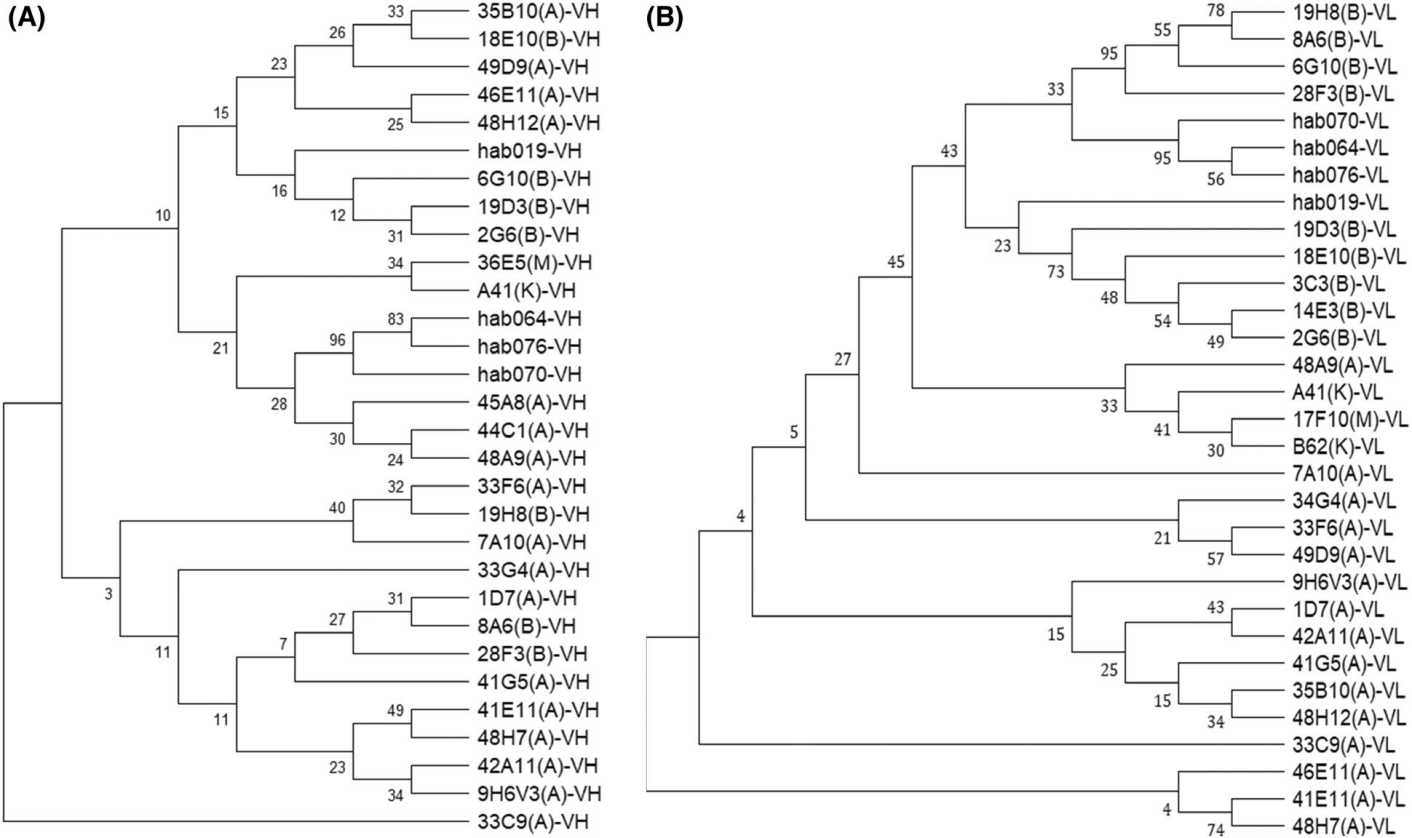
Evolutionary analysis between candidates and antibodies from patents. (A) Evolutionary analysis of the VH of lead candidates and patent antibodies; (B) evolutionary analysis of the VL of lead candidates and patent antibodies. The evolutionary trees were analyzed using MEGA X. A stands for the clone amino acid sequence from the Amgen patent anti‐GITR antibody (US9464139 B2), B stands for the clone amino acid sequence from the BMS patent anti‐GITR antibody (US9228016), M stands for the clone MK‐4166 amino acid sequence from Merck & Co. (Palo Alto, CA, USA), and Schering‐Plow (Kenilwroth, NJ, USA; US8709424B2), and K stands for the clone amino acid sequence from Korea National Cancer Centre patent anti‐GITR (US9255151B2).

Variable regions of chimeric antibodies were genetically fused to a human IgG1 kappa backbone as this majority IgG type can mediate strong effects and has desirable biophysical properties [26]. A summary of the characterization of these fully human anti‐GITR antibodies is in Table S1. Four antibodies, hab019, hab064, hab070, and hab076, were selected as lead candidates considering the potent activity in NF‐ĸB signal pathway and T‐cell activation, and also the uniqueness of antibody variable sequences. In an amino acid sequence alignment BLAST results, the sequence of the lead candidates displayed no more than 90% homology to antibodies in patents, (Table S2 and Fig. 1). From the tree, we conclude that the four antibodies (hab019, hab064, hab070, and hab076) had no infringement with patent antibodies.

With potential therapeutic antibodies, poor stability can result from deamidation, isomerization, glycation, and cyclization, among other modifications. The four lead candidates were thus engineered with modifications for manufacturability to delete translational modification hot spots so that they would be familiar with germline sequences. hab019 had one ‘DG’ in CDR‐H3 in VH, which can be converted from ‘DG’ to ‘EG’ or ‘DA’ due to the isomerization of DG [27, 28, 29, 30], Additionally, ‘SSVRP’ in FR3 in VH can be converted to ‘NSLRA’ without affecting its function based on a 3D model, which improves germline function in framework residues for reducing immunogenicity. Cys in FR3 in VL of other antibodies was converted to Tyr because Cys and N‐glycosylation sites need to be avoided during optimization [31], and Trp in FR2 in VL was converted to Lys due to being oxidation‐prone residues or hydrophobicity‐driven aggregation, both of which impact function [32, 33]. The mutation sites of the lead candidate antibodies are summarized in Table 1. The engineered antibodies were next produced in 293F cells. All of our results demonstrated that the four lead candidates were novel GITR antibody molecules.

**Table 1:** Summary of mutation sites of lead candidate antibodies. VH/VL amino acid sequences of antibodies are available in patent CN 111234018A. For example, the amino acid sequence of the heavy chain variable region of hab019 is seq ID no. 1 in the sequence table in this patent. The mutation sites are marked in brackets, NLA means ‘SSVRP’ is converted to ‘NSLRA’ in the amino acid sequence, and ‘EG’ or ‘DA’ is converted from ‘DG.’ ‘N2S’ indicates ‘N’ is converted to ‘S’ in the amino acid sequence. Other similar constructions have similar meanings.

Antibody code#	VH	VK
hab019	3503‐hab019‐VH	3503‐hab019‐Vk
	SEQ ID No. 1	SEQ ID No. 5
hab019e1	3503‐hab019‐VH(DG + NLA)	3503‐hab019‐Vk
	SEQ ID No. 41	SEQ ID No. 5
hab019e2	3503‐hab019‐VH(EG + NLA)	3503‐hab019‐Vk
	SEQ ID No. 42	SEQ ID No. 5
hab019e3	3503‐hab019‐VH(DA + NLA)	3503‐hab019‐Vk
	SEQ ID No. 44	SEQ ID No. 5
hab064e1	3503‐hab064‐VH(N2S)	3503‐hab064‐Vk(T2K/C2Y)
	SEQ ID No. 46	SEQ ID No. 47
hab070e1	3503‐hab070‐VH	3503‐hab070‐Vk(C2Y)
	SEQ ID No. 9	SEQ ID No. 48
hab076e1	3503‐hab076‐VH	3503‐hab076‐Vk(C2Y)
	SEQ ID No. 17	SEQ ID No. 49

### Characterization of the antigen‐binding activity of GITR antibodies

**Fig. 2:**
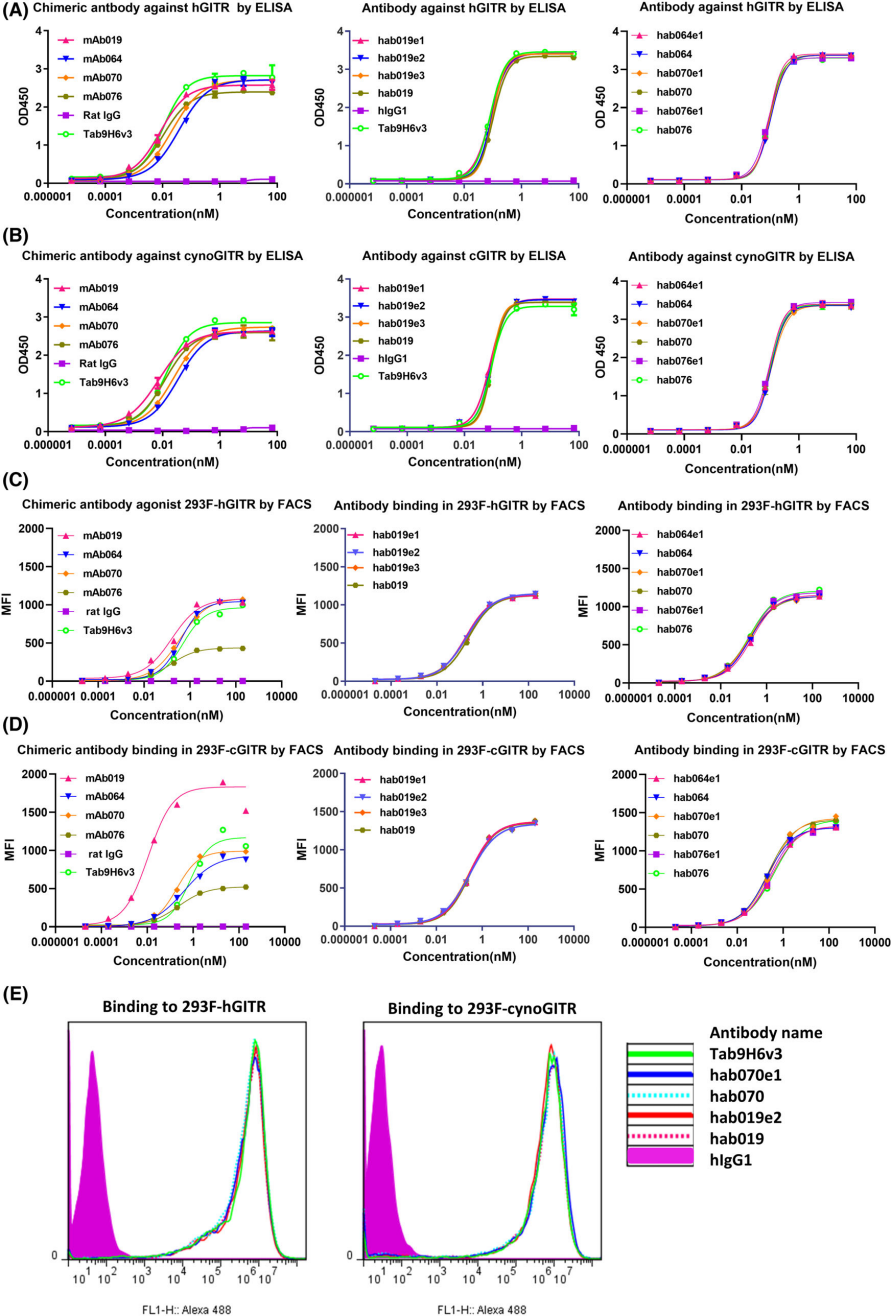
Binding activity of four lead candidate antibodies with human and cynoGITR. (A) the binding activity to human GITR of four candidate antibodies by ELISA. (B) the binding activity to cynoGITR of four candidate antibodies by ELISA. (C) the binding activity to 293F‐hGITR of four candidate antibodies by FACS. (D) the binding activity to 293F‐cynoGITR of four candidate antibodies by FACS. (E) the histogram of the representative antibodies binding to GITR overexpression cell by FACS at 200 nm. Tab9H6v3 was a positive control, while hIgG1 was an iso‐control. All error bars indicate the SEM. [Colour figure can be viewed at wileyonlinelibrary.com]

Due to the amino acid sequence homology of GITR protein in human and cynomolgus being as high as 88.09%, cynomolgus was used for preclinical pharmacokinetics and toxicology assessments related to GITR antibodies. Human/cynoGITR protein was employed to assess the binding properties of the four GITR antibodies by ELISA, and the result is shown in Fig. 2A,B. Along with this, 293F‐human/cynoGITR cells were employed to detect the binding properties by FACS, and the results are shown in Fig. 2C,D. The binding activity to GITR protein by these four fully human candidate antibodies and their modification antibodies were similar to Tab9H6v3 (an EC50 of 0.07095 nm for hGITR and an EC50 of 0.06369 nm for cynoGITR) by ELISA, and the binding activities showed a little difference between human‐murine chimeric antibodies and human antibodies, which may have been due to the different secondary peroxidase antibodies and isotypes. In cell‐based assays, the binding activity to 293F‐hGITR/cynoGITR of our four human candidate antibodies and their modifications were similar to Tab9H6v3 (an EC50 of 0.184 nm for 293F‐hGITR and an EC50 of 0.235 nm for 293F‐cynoGITR) by FACS, such as hab019 and hab070, hab019e2 and hab070e1 were consistent with Tab9H6v3 shown in Fig. 2E. And most of the binding activities for these were higher after humanization due to the different isotypes of these chimeric antibodies.

**Fig. 3:**
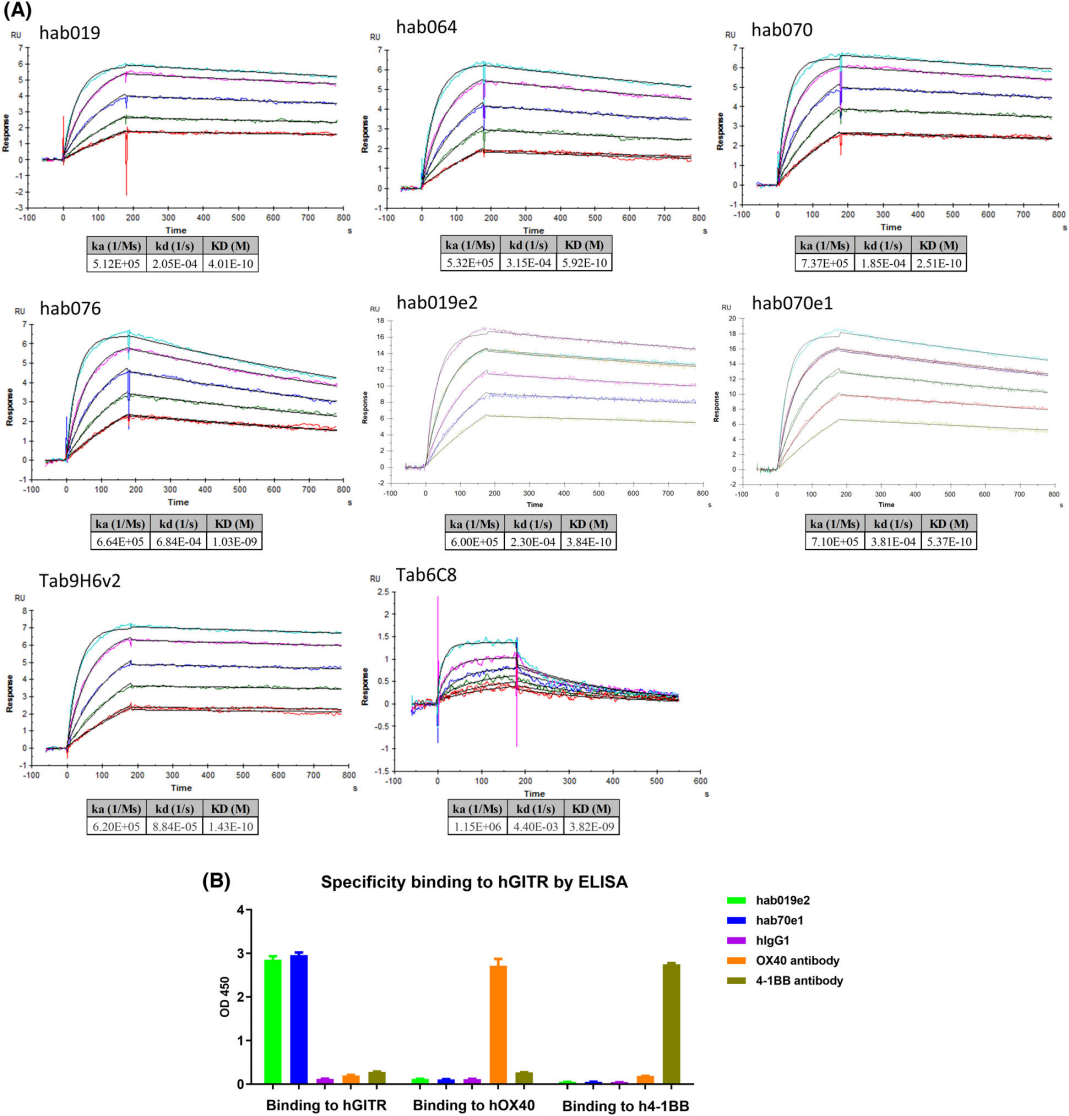
(A) The affinity of antibodies and hGITR‐ECD‐hFc detected by Biacore. Five concentrations of antibodies were 3.125, 6.25, 12.5, 25, and 50 nm. (B) Specificity of binding to hGITR by ELISA. ELISA plates were pre‐coated with human OX40 ECD hFc, 4‐1BB ECD hFc, or hGITR ECD hFc as an antigen at 1 μg·mL^−1^ overnight at 4 °C, and after washing and blocking, antibodies were added to the plates and detected by secondary antibodies with HRP. OX40 and 4‐1BB are TNFR family members and GITR's paralogues. XO40 and 4‐1BB antibodies were used as positive controls, and hIgG1 was a negative control. The data of ELISA are shown as the mean ± SEM. [Colour figure can be viewed at wileyonlinelibrary.com]

The binding results of antibodies in protein‐based and cell‐based assays were consistent with the affinity results detected by Biacore shown in Fig. 3A. The affinity between Tab6C8 and hGITR‐ECD‐hFc was much lower than the others. From these results, we could conclude that the fully human antibodies and engineering medication were consistent with their parent human‐murine antibodies, and their binding activities were similar to Tab9H6v3.

To accurately identify the specificity of binding activity to hGITR, ELISA was applied to analyze the affinities of hab019e2 and hab070e1 with GITR's paralogues (OX40 and 4‐1BB), and the results are shown in Fig. 3B. Importantly, hab019e2 and hab070e1 only bound specifically to human GITR and not to its paralogues (OX40 and 4‐1BB), indicating that hab019e2 and hab070e1 showed excellent binding specificity.

### Antibody induction of the NF‐ĸB signal pathway

**Fig. 4:**
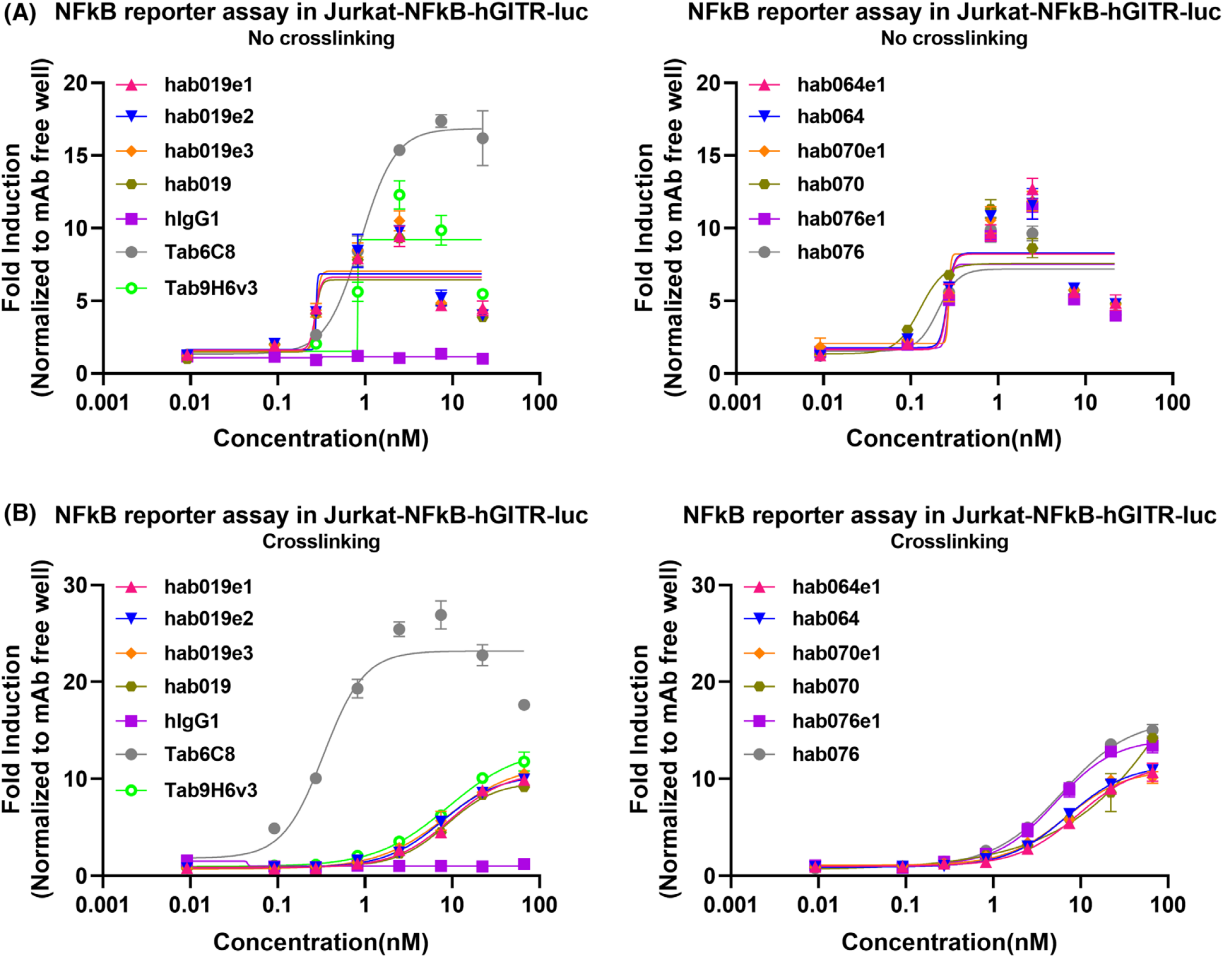
NF‐κB reporter assay in Jurkat‐NF‐κB‐Luc‐hGITR cell. Tab9H6v3 and Tab6C8 were used as positive controls, while hIgG1 was an iso‐control. (A) NF‐κB reporter assay in the no‐cross‐linking format. (B) NF‐κB reporter assay in the crosslinking format with anti‐hFc antibodies. All error bars indicate the SEM. Tab6C8 was much better than the other antibodies in both cross‐linking and non‐cross‐linking assay, **P < 0.001. Tab9H6v3 was similar to the four lead candidate antibodies, *P* > 0.05. One‐way ANOVA was used to determine statistical significance. [Colour figure can be viewed at wileyonlinelibrary.com]

To assess the capacity of anti‐hGITR antibodies to induce the NF‐κB signaling pathway *in vitro* [34], a Jurkat‐NF‐κB‐Luc‐hGITR cell line was used. Anti‐hGITR antibodies and F(ab)2 anti‐human IgG, Fcγ‐specific fragments were co‐incubated with cells for a cross‐linking assay, and the results as shown in Table 2 and Fig. 4. The fully human and engineering modification antibodies (EC50 < 0.27 nm) were better than Tab9H6v3 (EC50 = 0.787 nm) in terms of their ability to induce the NF‐κB signal pathway in a non‐cross‐linking assay. However, their EC50 was similar to our cross‐linking assay, and the value was approximately 8 nm. Tab6C8 (EC50 = 0.3362 nm, Top value = 32) was much better than all of them (EC50 > 5 nm, top value < 20), with a higher top ratio and a lower EC50 in our cross‐linking assay.

**Table 2:** Characteristics of candidate antibodies. mAb019, mAb064, mAb070, and mAb076 were human‐murine chimeric antibodies from hybridoma cell production, and hab019, hab064, hab070, and hab076 were fully human antibodies from chimeric antibody conversion, while the others were engineering modification antibodies. Tab9H6v3 and Tab6C8 were used as positive controls, and hIgG1 was a negative control. In the NF‐ĸB luciferase reporter assay, the stimulation fold was the ratio of antibody RLU values related to that of the antibody‐free well. In the T‐cell activation assay, the EC50 of IFN‐γ secretion was measured using an ELISA kit from R&D. A slash (/) means not applicable, and (−) means not tested.

Antibody code#	ELISA (OD450)		FACS (MFI)		NF‐ĸB luciferase reporter assay				T‐cell activation				Affinity by Biacore
	hGITR	cGITR	293F‐hGITR	293F‐cGITR	No crosslinking		Crosslinking		Donor#1	Donor#2	Donor#3	Donor#4	
	EC50 (nm)	EC50 (nm)	EC50 (nm)	EC50 (nm)	EC50 (nm)	Top ratio	EC50 (nm)	Top ratio	EC50 (nm)	EC50 (nm)	EC50 (nm)	EC50 (nm)	K_*D*_ (m)
mAb019	0.008	0.007	0.163	0.011	N/A	20	12.49	11	–	–	–	–	–
mAb064	0.038	0.035	0.385	0.404	> 0.20	10	0.101	7	–	–	–	–	–
mAb070	0.022	0.025	0.358	0.198	0.062	10	> 0.09	7	–	–	–	–	–
mAb076	0.01	0.012	0.152	0.226	> 0.09	14	> 0.27	8	–	–	–	–	–
hab019	0.103	0.092	0.244	0.304	0.265	10	8.664	13	0.00057	0.00028	0.00047	0.00667	4.01E‐10
hab064	0.106	0.108	0.165	0.22	0.177	10	7.126	12	0.00070	0.00073	0.00046	0.00232	5.92E‐10
hab070	0.099	0.106	0.19	0.19	0.111	9	7.324	16	0.00098	0.00152	0.00051	0.00084	2.51E‐10
hab076	0.091	0.101	0.179	0.418	0.157	9	6.067	20	0.00069	0.00220	0.00050	0.00064	1.03E‐09
hab019e1	0.082	0.079	0.181	0.287	0.26	8	9.462	12	–	–	–	0.00698	–
hab019e2	0.093	0.089	0.206	0.29	0.275	8	7.688	13	–	–	–	0.00343	3.84E‐10
hab019e3	0.098	0.089	0.215	0.277	0.269	9	8.75	14	–	–	–	0.00790	–
hab064e1	0.093	0.096	0.235	0.313	0.264	11	9.255	12	–	–	–	0.00236	–
hab070e1	0.101	0.111	0.164	0.268	0.262	7	6.775	12	–	–	–	0.00836	5.37E‐10
hab076e1	0.087	0.094	0.195	0.26	0.264	8	5.164	13	–	–	–	0.00094	–
Tab9H6v3	0.088	0.09	0.184	0.235	0.787	11	8.863	14	0.00068	0.00069	0.00086	0.00461	1.35E‐10
Tab6C8	0.954	1.125	0.027	0.007	0.829	14	0.3362	32	0.01064	0.00559	0.00681	–	3.82E‐09
hIgG1	/	/	/	/	/	/	/	/	/	/	/	/	/

### Functional T‐cell activation

**Fig. 5:**
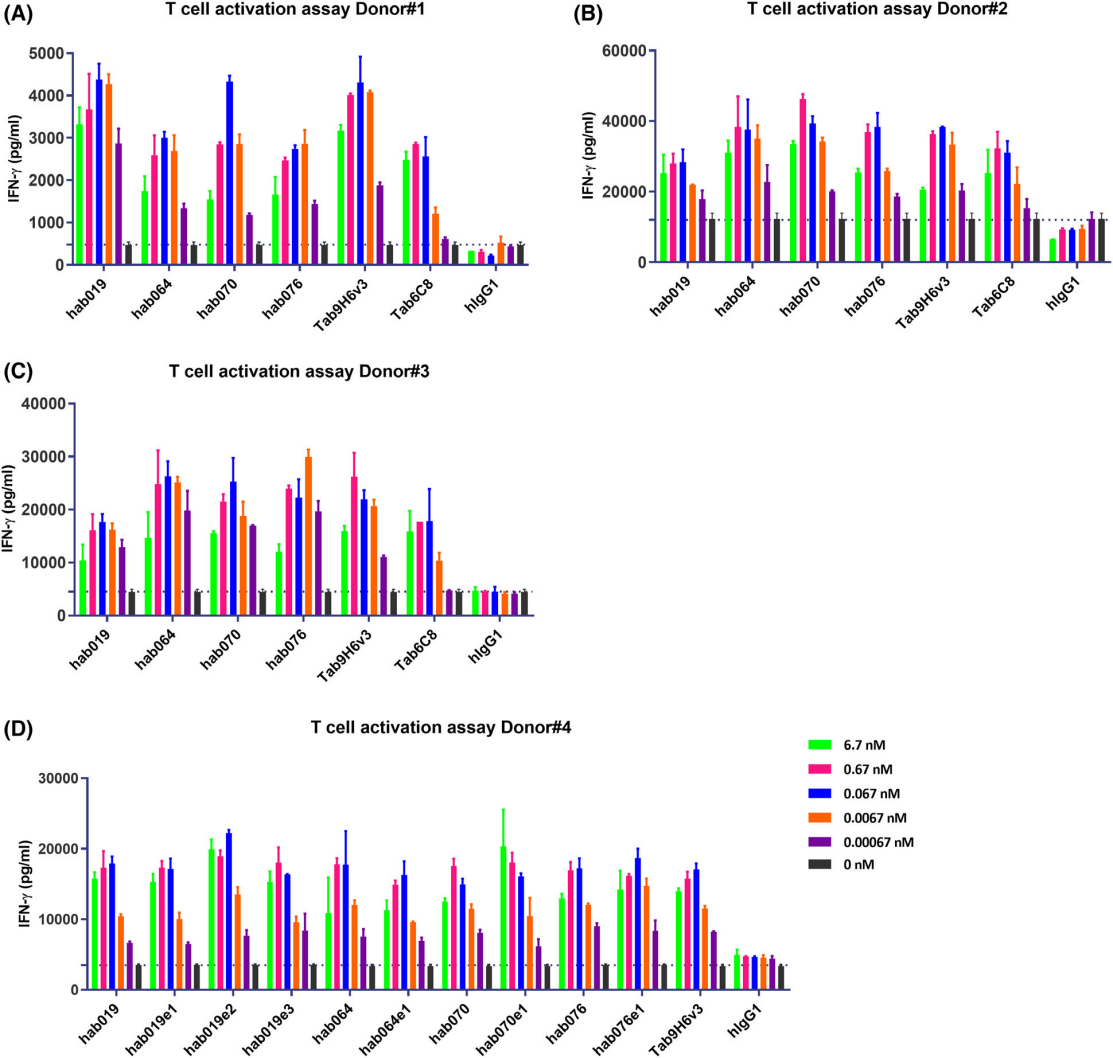
Lead candidate antibodies show activation of T cells by IFN‐γ production. (A–C) the four lead candidate antibodies all strongly promoted T‐cell activation in a dose‐dependent manner in three different PBMC donors (donor #1, donor #2, and donor #3). (D) Engineering modification antibodies behaved consistently to antibodies before modification in promoting T‐cell activation in donor #4. In (A–D) human total T cells were activated by GITR antibody (with pre‐coated 0.3 μg·mL^−1^ anti‐CD3 antibody and 2.7 μg·mL^−1^ anti‐hFc antibody), and then IFN‐γ secretion in culture medium was detected by ELISA. Each assay was performed in duplicate, and the data shown are the mean ± SEM. [Colour figure can be viewed at wileyonlinelibrary.com]

As it is a critical property of an efficacious antibody, the T‐cell activation activity of the lead candidate antibodies was measured [34]. T‐cell activation requires two distinct signals, the first signal depends on the ligation of the T‐cell receptor (TCR)/CD3 complex and the CD4 or CD8 coreceptors, which can be activated by an anti‐CD3(OKT3) antibody [35, 36]. The second signal can be provided by cell surface molecules that mediate essential co‐stimulatory signals, thereby complementing the TCR/CD3‐mediated events such as GITR, and the ligation of GITR with agonistic antibodies and anti‐hFc antibody cross‐linking synergizes with TCR‐mediated signaling to initiate and maintain T‐cell responses [36, 37]. The augmentation of T‐cell activation was further confirmed in terms of cytokine production, such as IFN‐γ [36]. We also applied three different donors to exclude the effects of individual differences, as shown in Fig. 5A–C. Dose‐dependent response of antibody candidates was observed within the range of 0.00067–6.7 nm, and IFN‐γ production ranged from 5000 to 60 000 pg·mL^−1^ in different donors, while the EC50 values of the induced index activation by Tab9H6v3 were similar in the four different donors (EC50 range from 0.0006 to 0.0009 nm). Moreover, the EC50 of hab019 ranged from 0.0004 to 0.0006 nm and the EC50 of hab070 ranged from 0.0005 to 0.0015 nm, which was similar to that of Tab9H6v3.

Engineering modification antibodies were consistent with their parent antibodies in terms of T‐cell activation, and modification did not affect the activation of T cells in Donor #4, as is shown in Fig. 5D. hab019e2 (EC50 = 0.00343 nm) and hab070e1 (EC50 = 0.00836 nm) showed similar potency in T‐cell activation assays as Tab9H6V3 (EC50 = 0.0641 nm). Based on the biochemical behavior and functional outcome, we concluded that hab019e2 and hab070e1 were potential effective therapeutic antibodies worthy of further in‐depth investigations.

### Binding activity with humanized B‐hGITR mice spleen cells

**Fig. 6:**
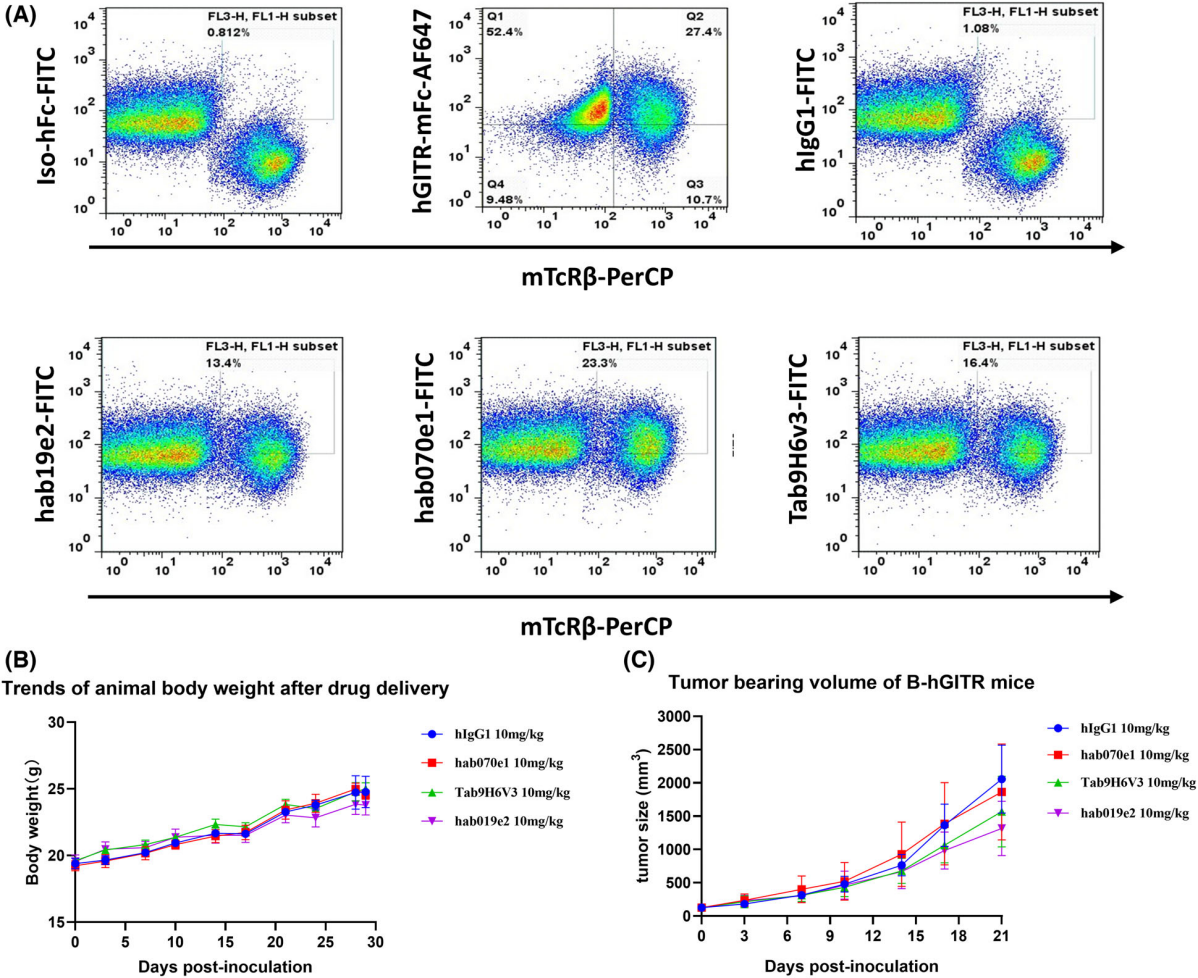
(A) Binding activity of hab019e2, hab070e1, and Tab9H6v3 *in vivo*. Splenocytes were collected from homozygous B‐hGITR mice stimulated with anti‐CD3 antibody (7.5 μg per mice) *in vivo*, and the cells were prepared in suspension and stained by fluorescence‐labeled antibodies, then analyzed by flow cytometry. The iso‐hFc and hIgG1 were negative controls, and hGITR‐mFc antibody was the positive control. (B) Body weight changes of mice monitored three times a week during the trial. The drug volume was calculated according to the body weight of each experimental animal at 10 μL·g^−1^. Error bars indicate the SEM (*n* = 6). (C) Tumor‐bearing volume (mm^3^) changes of mice were monitored three times a week. On day 29 after mice received tumor cells, all mice were killed, and tumors were collected and weighed (in Table 2). The calculation formula of tumor volume was as follows: Tumor volume = 0.5 × long diameter × short diameter^2^. One way‐ANOVA was used to analyze statistical significance. Error bars indicate the SEM (*n* = 6). [Colour figure can be viewed at wileyonlinelibrary.com]

Due to the relatively low homology between mGITR and hGITR, the hGITR antibody could only bind to hGITR protein at high affinity compared with mGITR protein. Human GITR gene knock‐in C57BL/6 mice, B‐hGITR mice is an ideal humanized mice model for assessing the antitumor effect of our antibodies. For *in vivo* binding assay, B‐hGITR mice were treated with anti‐CD3 antibody to induce hGITR expression [36], and then hGITR expression was detected using lead candidate hGITR antibodies by FACS after 24 h. The results of FACS dot plots are shown in Fig. 6A, and the data indicated that hab019e2, hab070e1, and Tab9H6V3 all showed binding activity to human GITR in spleen cells compared with hIgG1 control. B‐hGITR mice would be a suitable mice model for evaluating the potent antitumor immunity of human GITR antibodies.

### *In vivo* efficacy evaluation in B‐hGITR MC38 transplanted mice

To evaluate the effect(s) of target engagement, intraperitoneal (IP) administration of drugs in experimental studies involving rodents is a justifiable route for pharmacological and proof‐of‐concept studies [38]. Using the mouse MC38 tumor system, B‐hGITR mice were then used to evaluate the antitumor activity of our antibodies *in vivo* with I.P injection. The mice were weighed initially, as shown in Fig. 6B and Table 3. All experimental animals had good activity and feeding status during the entire administration period, and their body weight increased to a certain extent. There were no significant differences in animal body weight between the test group and the iso‐control group at 21 days after administration (*P* > 0.05). As animal weight as a direct aspect may reflect overall toxicity, we hypothesized that hab19e2 and hab070e1 were well tolerated and had strong safety features in B‐hGITR mice. Tumor size was measured twice with a vernier caliper and recorded in Fig. 6C and Table 3. After 21 days of the administration, the TGI% of the hab070e1 group and the Tab9H6v3 group were 10.0% and 25.3%, respectively, compared to the hIgG1 control group. The results revealed Tab9H6v3 and hab070e1 had failed to inhibit tumor growth (*P* > 0.05). In contrast, the TGI% of the hab019e2 group was 38.3%, indicating that hab019e2 had a significant inhibitory effect on tumor volume growth, compared with the hIgG1 control group (*P* < 0.05). In addition, the average tumor weight of the Tab9H6v3 group and the hab019e2 group decreased to a certain extent compared to the hIgG1 control group, indicating that hab019e2 and Tab9H6v3 inhibited MC38 tumor weight growth similarly (*P* > 0.05).

**Table 3:** Efficacy test of hab019e2 and hab070e1 in B‐hGITR mice bearing MC38 tumors.

Group	Number of animals	Tumor volume (mm^3^)^a^				Tumor weight (g)^a^	
		Before dose	21 days after dose	TGI (%)	*P* ^b^	29 days after dose	*P* ^b^
hIgG1	6	126 ± 6	2056 ± 208	–	–	5.229 ± 0.652	–
Tab9H6v3	6	126 ± 6	1569 ± 217	25.3	0.136	4.261 ± 0.337	0.22
hab70e1	6	126 ± 5	1864 ± 294	10	0.605	5.909 ± 0.819	0.53
hab19e2	6	126 ± 5	1317 ± 167	38.3	0.019	3.824 ± 0.449	0.11

^a^ Mean ± standard error.

^b^ Statistical comparison of tumor volume or weight between the drug administration group and the solvent control group after 21 or 29 days assessed by Student's *t*‐test. Comparison of tumor weight in each group after 29 days of administration by Student's *t*‐test.

## Discussion

In this project, we immunized H2L2 mice with different strategies (DNA, protein, and cell immunization) to generate GITR antibodies. H2L2 mice showed strong immune responses via all of these strategies except for gene immunization, and the mice with the highest titers were selected for fusion to generate human‐murine chimeric antibodies through hybridoma technology. These anti‐hGITR antibodies were then screened by FACS binding and NF‐ĸB reporter assays. In NF‐ĸB reporter assays, Tab6C8 showed higher activity than fully human antibodies from the H2L2 mice and Tab9H6v3, which may be because that Tab6C8 is a fully humanized Fc‐dysfunctional aglycosylated IgG1κ monoclonal antibody. But this is not consistent with the results of T‐cell activation. Tab9H6v3, a fully hIgG1 from XenoMouse(R) mice, was more potent than Tab6c8 in T‐cell activation, and the data of primary T‐cell activation may be more relevant to *in vivo* antitumor efficacy, so Tab9H6v3 could be a more potent positive control *in vivo* experiments. hab019e2 and hab070e1 were outstanding in terms of T‐cell activation with neo‐sequence in VL and VH, and were selected for *in vivo* experiments. Moreover, the antibody hab019e2 resulted in significantly inhibited tumor growth in MC38 tumor bearing mice. We hypothesize that hab019e2 may stimulate GITR on effector T cells resulting in high‐avidity T‐cell responses that were tumor‐specific *in vivo* just like we saw in the *in vitro* T‐cell activation experiment. Therefore, hab019e2 may have more potent antitumor effects *in vivo* than Tab9H6v3.

Most of the antibodies obtained from cell immunization exhibited more potent agonist activities *in vitro* functional activity assays. The serum antibody titers in mice immunized with DNA were much lower than that in other groups, indicating the poor expression of hGITR in mice *in vivo* after DNA injection. Cell immunization could induce the production of more functional antibodies than protein immunization, although protein immunization also resulted in high serum antibody titers. It may have been that hGITR immunogen overexpression on the 293F‐hGITR cells was likely to adopt its native configuration.

We obtain fully human antibodies by converting rat IgG Fc to human IgG1 Fc because the variable region of the antibodies produced in H2l2 mice is completely anthropogenic, and this had a decisive influence on the specific function and properties of these antibodies. This reduced the risk of functional decline or loss during humanization and modification in variable regions, compared with antibody production from immunizing conventional mice. This allowed making subsequent modifications faster and easier, with a lower risk of failure, which saved time and costs in our research. hab019e2 was obtained with high affinity (K_*D*_ = 3.84 × 10^−10^ m) and showed strong T‐cell activation. These novel properties of hab019e2 suggest that it may be a better candidate antibody than others in the field, and further research on this is suggested as compared to other anti‐hGITR antibodies. Moreover, after translational modification hot spot removal and consistent with germline sequences, hab019e2 appears to have better developability.

Targeting of GITR in mice has been shown to promote potent antitumor immunity in a range of models through the activation of T cells and the localized modulation and/or depletion of intratumoral Tregs. For MK‐4166, a humanized anti‐hGITR antibody, its murine surrogate mAb(DTA‐1) was used in 4T1 and CT26 tumor models, and the data indicated DTA‐1 alone exhibited no tumor rejection while DTA‐1 combined with anti‐TGF‐β or irradiation induce the synergistic antitumor efficacy [15], and responses were only seen with combination treatment in clinical trials [17]. TRX‐518 (also known as Tab6C8 in the manuscript), its murine surrogate mAb was shown effective in the B16 melanoma model with monotherapy (6C8, Leap Therapeutics, Cambridge, MA, USA; US20170137527 A1). TRX518 monotherapy and in combination showed limited clinical responses associated with immune activation in clinical studies [39]. Mouse GITR has been shown to signal effectively as a dimer while, in contrast, human GITR requires trimerization [40, 41, 42]. Given this difference, we reasoned that there might be a discrepancy of mechanism between human and mouse GITR. Despite GITR agonist antibodies were demonstrated therapeutic potential in many preclinical models, data from clinical studies suggest that GITR agonist antibodies may not be sufficient to produce a robust anticancer effect in a heavily pretreated population in monotherapy and combination therapy with other immune checkpoint inhibitors may further increase the overall response rate. Our antibody candidate hab019e2 showed an antitumor response in B‐hGITR mice model as monotherapy, and it will be more interesting to see how it performs under a combination therapy setting.

In summary, our research to get fully human antibodies against GITR was aided greatly using immunization of H2L2 mice and the creation of a hybridoma platform. Our findings show that more functional clones were generated by cell immunization. Meanwhile, the desired antibodies had consistent affinity and function between human‐murine chimeric antibodies and their derived fully human antibodies with engineering modification. As they showed outstanding performance in antitumor effects *in vivo*, the neo‐sequence with hot spot removed and germline mutation of hab019e2 was different from existing antibodies in patents, and it is expected to be different or even better than existing antibodies in subsequent clinical trials. hab019e2 thus is a promising fully human candidate antibody for tumor immunotherapy.

## Conflict of interest

QT, TY, HL, and QQ are current employees of ChemPartner. CD are current employees of Shanghai PharmaExplorer. Patent pertaining to the results presented in this paper have been filed by Shanghai PharmaExplorer (Publication Number CN 111234018 A). The other author declares no conflict of interest.

## Author contributions

TY, HL, and CD conceived and designed the project. QT and QQ performed the experiments and analyzed the data. QT wrote the manuscript. FQ, HL, and TY made manuscript revisions. All authors read and approved the final manuscript.

## Supporting information


**Table S1.** Summary of 42 fully human GITR antibodies (from human‐murine chimeric antibodies to human antibodies). Tab6C8 and Tab9H6v3 were reference antibodies, and hIgG1 was used as an isotype control. In NF‐ĸB reporter assays, Max‐window is the ratio of luminescence normalized to that of the 0 nM well. In T‐cell activation assays, the EC50 of IFN‐γ secretion refers to the capacity of T‐cell activation. All of the data were analyzed by GraphPad. A slash (/) means insignificant data were obtained. (+) very slightly, (++) slightly, and (+++) activation, (−) N/A.

**Table S2.** Summary of BLAST results of candidates with patent antibody sequences. Max_Identity of VH or VH between the candidates and patent antibodies was analyzed.

## Data Availability

The data in the project are available from the corresponding author upon reasonable request.
