# Supplementary material for: Development of a fully human anti‐GITR antibody with potent antitumor activity using H2L2 mice
Source: FEBS Open Bio. 2022 Jun 21;12(8):1542–57. doi: 10.1002/2211-5463.13451 (PMC9340783; doi:10.1002/2211-5463.13451)
Supplement: Supplementary file 1 — Table S1. Summary of 42 fully human GITR antibodies (from human‐murine chimeric antibodies to human antibodies). Tab6C8 and Tab9H6v3 were reference antibodies, and hIgG1 was used as an isotype control. In NF‐ĸB reporter assays, Max‐window is the ratio of luminescence normalized to that of the 0 nM well. In T‐cell activation assays, the EC50 of IFN‐γ secretion refers to the capacity of T‐cell activation. All of the data were analyzed by GraphPad. A slash (/) means insignificant data were obtained. (+) very slightly, (++) slightly, and (+++) activation, (−) N/A. [file FEB4-12-1542-s001.docx]

**Table S1.** Summary of 42 fully human GITR antibodies (from human-murine chimeric antibodies to human antibodies). Tab6C8 and Tab9H6v3 were reference antibodies, and hIgG1 was used as an isotype control. In NF-ĸB reporter assays, Max-window is the ratio of luminescence normalized to that of the 0 nM well. In T cell activation assays, the EC50 of IFN-γ secretion refers to the capacity of T-cell activation. All of the data were analyzed by GraphPad. A slash (/) means insignificant data were obtained. (+) very slightly, (++) slightly, and (+++) activation, (–) N/A.

| Antibody Code# | Clone# | | Antigen | Isotype | FACS binding | | |  | NF-ĸB reporter assay | | | | T Cell Activation  EC50 (nM) |
| --- | --- | --- | --- | --- | --- | --- | --- | --- | --- | --- | --- | --- | --- |
|  |  |  |  |  | 293F-hGITR | | 293F-cGITR |  | Non-crosslinking | | Crosslinking | |  |
|  |  |  |  |  | EC50(nM) | | EC50 (nM) |  | EC50 (nM) | Top ratio | EC50 (nM) | Top ratio |  |
| hab002 | 2G8B1D6 | hGITR-ECD-hFc | | IgG2b,κ | | 1.065 | 0.197 |  | 1.105 | 12 | 10.76 | 7 | 0.0066 |
| hab003 | 37B10B3 | hGITR DNA +hGITR-ECD-hFc | | IgG1,κ | | 0.044 | 0.075 |  | / | / | 17.82 | 11 | +++ |
| hab005 | 666/1G4 | hGITR-ECD-hFc | | IgM,κ | | 0.156 | 0.433 |  | >0.82 | 9 | 20.4 | 4 | 0.0007 |
| hab006 | 14D4B11 | hGITR DNA + hGITR-ECD-hFc | | IgM,κ | | / | / |  | / | / | / | / | / |
| hab007 | 35H4G4 | hGITR DNA + hGITR-ECD-hFc | | IgM,κ | | 0.089 | 0.033 |  | 0.363 | 10* | 17.91 | 5 | ++ |
| hab008 | 74H5F4 | Rena-hGITR & 293F-hGITR | | IgM,κ | | + | / |  | / | / | / | / | / |
| hab009 | 87D11D10 | Rena-hGITR & 293F-hGITR | | IgM,κ | | / | 0.11 |  | 7.07 | 16 | 1.228 | 14 | + |
| hab010 | 90H3E6 | Rena-hGITR & 293F-hGITR | | IgM,κ | | / | / |  | / | / | / | / | / |
| hab011 | 95A4B2 | Rena-hGITR & 293F-hGITR | | IgM,κ | | / | / |  | / | / | / | / | / |
| hab012/1 | 98F10A6 | Rena-hGITR & 293F-hGITR | | IgM,κ | | 0.45 | 0.22 |  | 14.49 | 16 | 5.743 | 6 | 0.0077 |
| hab012/2 | 98F10A6 | Rena-hGITR & 293F-hGITR | | IgM,κ | | / | / |  | / | / | / | / | / |
| hab013 | 82E11B11 | Rena-hGITR & 293F-hGITR | | IgG2b,κ | | 0.02 | / |  | 0.823 | 10 | 46.49 | 6 | +++ |
| hab014 | 85E2F12 | Rena-hGITR & 293F-hGITR | | IgG2b,κ | | 0.069 | 0.033 |  | 0.274 | 11 | 42.57 | 9 | +++ |
| hab019 | 96A10H9 | Rena-hGITR & 293F-hGITR | | IgG2b,κ | | 0.226 | 1.267 |  | >0.27 | 5 | 26.31 | 7 | +++ |
| hab020 | 137F10D12 | hGITR-ECD-hFc | | IgG2b,κ | | 0.167 | 0.324 |  | / | / | 2.78 | 15 | +++ |
| hab022 | 127F6H2 | hGITR-ECD-hFc | | IgG2b,κ | | 0.2 | 0.243 |  | >0.27 | 7 | 30.13 | 8 | +++ |
| hab027 | 156F3F3 | 293F-hGITR | | IgM,κ | | 0.14 | 0.45 |  | 17.57 | 9 | 29.09 | 4 | / |
| hab029 | 171108 | 293F-hGITR | | IgM,κ | | / | / |  | / | / | / | / | / |
| hab031 | 191E7D6 | hGITR-ECD-hFc | | IgM,κ | | 0.06 | 2.12 |  | 2.129 | 9 | 10.65 | 6 | 0.0103 |
| hab032 | 193A7B12 | hGITR-ECD-hFc | | IgM,κ | | 0.55 | 1.76 |  | 34.09 | 5 | / | / | 0.0054 |
| hab033 | 193E8E7 | hGITR-ECD-hFc | | IgM,κ | | 0.52 | 0.58 |  | 12.2 |  | 3 | /  / | 0.0005 |

**Table S1.** (continued)

| Antibody Code# | Clone# | Antigen | Isotype | FACS binding | |  | NF-ĸB reporter assay | | | | T CellActivation EC50 (nM) |
| --- | --- | --- | --- | --- | --- | --- | --- | --- | --- | --- | --- |
|  |  |  |  | 293F-hGITR | 293F-cGITR |  | Non-crosslinking | | Crosslinking | |  |
|  |  |  |  | EC50(nM) | EC50 (nM) |  | EC50 (nM) | Top ratio | EC50 (nM) | Top ratio |  |
| hab034 | 194H2F2 | hGITR-ECD-hFc | IgM,κ | 0.08 | 0.14 |  | >0.27 | 6 | 2.254 | 6 | 0.0002 |
| hab035 | 195G1G6 | hGITR-ECD-hFc | IgM,κ | 0.36 | 0.31 |  | / | / | / | / | 0.0035 |
| hab037 | 196E4B4 | hGITR-ECD-hFc | IgM,κ | 0.04 | 0.08 |  | >0.82 | 13 | 10.54 | 5 | 0.0002 |
| hab051 | 217G4G4 | hGITR-ECD-hFc | IgM,κ | 0.15 | 0.28 |  | 0.491 | 8 | 12.75 | 6 | ++ |
| hab052 | 212F8D7 | hGITR-ECD-hFc | IgM,κ | 0.7 | / |  | / | / | / | / | / |
| hab054 | 221F12H9 | hGITR-ECD-hFc | IgM,κ | 0.04 | 0.06 |  | 0.168 | 7 | 1.439 | 6 | +++ |
| hab055 | 227B54 | hGITR-ECD-hFc | IgM,κ | 0.08 | 0.11 |  | >0.27 | 7 | 19.54 | 7 | 0.0003 |
| hab059 | 253E6/1G9 | hGITR-ECD-hFc | IgM,κ | 39.96 | 37.35 |  | >0.82 | 6 | 29.69 | 3 | / |
| hab060 | 2112G8 | hGITR-ECD-hFc | IgM,κ | 0.52 | / |  | / | / | / | / | / |
| hab061 | 261E1G6 | 293F-hGITR | IgG2b,κ | 0.134 | 0.227 |  | / | / | 6.942 | 10 | +++ |
| hab062 | 264A111 | hGITR-ECD-hFc | IgM,κ | / | / |  | / | / | / | / | / |
| hab063 | 264B1E1 | 293F-hGITR | IgG2b,κ | 0.126 | / |  | / | / | / | / | +++ |
| hab064 | 265G9A11 | 293F-hGITR | IgG2b,κ | 0.068 | 0.133 |  | / | / | 9.593 | 13 | +++ |
| hab070(VL-1) | 272G5E1 | 293F-hGITR | IgG2b,κ | 0.04 | 0.029 |  | / | / | / | / | 0.0022 |
| hab070 | 272G5E1 | 293F-hGITR | IgG2b,κ | 0.04 | 0.029 |  | / | / | / | / | 0.0022 |
| hab073 | 274G9E11 | 293F-hGITR | IgG2b,λ | 0.011 | 0.005 |  | / | / | / | / | 0.0005 |
| hab076 | 27712G4 | 293F-hGITR | IgG2b,λ | 0.162 | 0.14 |  | / | / | / | / | +++ |
| hab096 | 339A34 | hGITR-ECD-hFc | IgM,κ | 0.071 | 0.181 |  | >0.82 | 13 | >7.41 | 5 | +++ |
| hab098 | 350B3B2 | hGITR-ECD-hFc | IgM,κ | 0.587 | 1.98 |  | >0.82 | 12 | >7.41 | 11 | 0.0003 |
| hab099 | 354B12G2 | hGITR-ECD-hFc | IgM,κ | 0.781 | 1.596 |  | >0.82 | 13 | >7.41 | 11 | 0.0003 |
| hab100 | 355H3F9 | hGITR-ECD-hFc | IgM,κ | 2.833 | 5.141 |  | >2.47 | 13 | >7.41 | 5 | 0.0065 |
| hIgG1 | – | – | hIgG1 | / | / |  | / | / | / | / | / |
| Tab6C8 | – | – | hIgG1 | 0.027 | 0.007 |  | 0.75 | 15 | 0.23 | 20 | / |
| Tab9H6v3 | – | – | hIgG1 | 0.19 | 0.16 |  | 0.38 | 6 | 4.86 | 6 | 0.0038 |
