# Supplementary material for: Development of a fully human anti‐GITR antibody with potent antitumor activity using H2L2 mice
Source: FEBS Open Bio. 2022 Jun 21;12(8):1542–57. doi: 10.1002/2211-5463.13451 (PMC9340783; doi:10.1002/2211-5463.13451)
Supplement: Supplementary file 2 — Table S2. Summary of BLAST results of candidates with patent antibody sequences. Max_Identity of VH or VH between the candidates and patent antibodies was analyzed. [file FEB4-12-1542-s002.docx]

**Table S2.** Summary of BLAST results of candidates with patent antibody sequences. Max_Identity of VH or VH between the candidates and patent antibodies was analyzed.

| Patent antibody | | | | Max_Identity | | | | | | | | | | |
| --- | --- | --- | --- | --- | --- | --- | --- | --- | --- | --- | --- | --- | --- | --- |
|  | |  | | hab019 | |  | hab064 | |  | hab070 | |  | hab076 | |
| Name | Company | | | VH | VL |  | VH | VL |  | VH | VL |  | VH | VL |
| TRX518 | | | GITR, Leap Thera, Tolerx | 55% | 69% |  | 54% | 79% |  | 54% | 67% |  | 55% | 64% |
| Amgen patent anti-GITR | | | Amgen | 90% | 88% |  | 90% | 88% |  | 88% | 89% |  | 90% | 89% |
| BMS patent anti-GITR | | | BMS | 90% | 92% |  | 87% | 91% |  | 86% | 92% |  | 88% | 92% |
| Korea patent anti-GITR | | | Korea NCC | 83% | 79% |  | 83% | 79% |  | 83% | 80% |  | 85% | 81% |
| Sanofi patent anti-GITR | | | Sanofi | 74% | 72% |  | 77% | 69% |  | 76% | 69% |  | 78% | 69% |
| FPA154 | | | Five Prime, Inhibrx | 77% | 35% |  | 68% | 31% |  | 68% | 35% |  | 69% | 32% |
| MK-4166 | | | Merck & Co, Schering-Plough | 78% | 82% |  | 82% | 82% |  | 82% | 83% |  | 83% | 81% |
| Dana Farber patent anti-GITR(WO) | | | Dana-Farber | 56% | 67% |  | 54% | 65% |  | 56% | 65% |  | 57% | 65% |
| INCAGN1876(WO) | | | 4-Antibody, Agenus, Incyte, Ludwig Inst., Sloan-Kettering | 59% | 69% |  | 53% | 65% |  | 53% | 67% |  | 53% | 68% |
| Novartis patent anti-GITR(WO) | | | Novartis | 74% | 72% |  | 71% | 71% |  | 73% | 71% |  | 74% | 71% |
